# Drohende ösophago-arterielle Fistel nach Batterieingestion – weltweit erste präventive Operation bei einem Kleinkind

**DOI:** 10.1007/s00101-024-01477-3

**Published:** 2025-01-08

**Authors:** Judith Lohmann, Tobias Klein, Martin Stenzel, Marko Aleksic, Paul Fuchs, Thomas Boemers, Jost Kaufmann

**Affiliations:** 1 Klinik für Kinder- und Jugendchirurgie und -urologie, Kinderkrankenhaus der Kliniken der Stadt Köln gGmbH, Amsterdamer Straße, Köln, Deutschland; 2 Radiologische Abteilung, Kinderkrankenhaus der Kliniken der Stadt Köln gGmbH, Amsterdamer Straße, Köln, Deutschland; 3 Klinik für Viszeral‑, Tumor‑, Transplantations- und Gefäßchirurgie, Krankenhaus der Kliniken der Stadt Köln, Merheim, Köln, Deutschland; 4 Klinik für Plastische und Ästhetische Chirurgie, Krankenhaus Merheim, Köln, Deutschland; 5 Fakultät für Gesundheit, Universität Witten/Herdecke, Witten, Deutschland; 6 Abteilung für Kinderanästhesiologie und Endoskopie, Kinderkrankenhaus der Kliniken der Stadt Köln gGmbH, Amsterdamer Str. 59, 50735 Köln, Deutschland

## Anamnese

Ein 3‑jähriges Kleinkind wurde in einer Notfallambulanz vorgestellt, weil es kurz zuvor eine Knopfzellbatterie verschluckt hatte. Das Kind habe danach eine Zeit lang gehustet und war dann beschwerdefrei. Bei einer HNO-ärztlichen Spiegelung des Larynx konnte kein Fremdkörper dargestellt werden. Das Kleinkind wurde etwa 3 h nach der Ingestion in unser Kinderkrankenhaus verlegt.

## Erstmaßnahmen

Das Mädchen präsentierte sich vollständig beschwerdefrei. Aufgrund der beobachtet verschluckten Knopfzellbatterie wurden sofort 10 ml Sucralfat-Suspension, 10 %ig, p.o. verabreicht und ein Röntgenbild mit Darstellung der Knopfzellbatterie im oberen Ösophagussphinkter angefertigt. Es erfolgte das sofortige Bergen der Knopfzelle mit einer Magill-Zange unter Darstellung mit einem Videolaryngoskop in tiefer Sedierung [1]. Hierbei zeigten sich ausgeprägte Verätzungen des Ösophagus.

## Verlauf

**Abb. 1:**
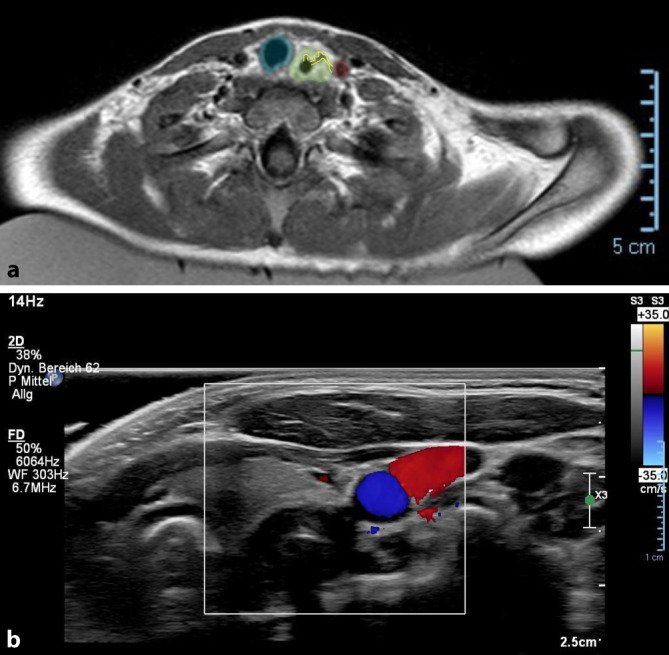
**a** MRT präoperativ. Trachea (*blau*), ödematöser Ösophagus (*grün*) mit 2 gangbildenden Ulzerationen (*gelb*), von denen einer bis in die Nähe der ACC (*rot*) reicht. **b** Farbkodierte Duplexsonographie: Ösophaguswand ohne erkennbare trennende Strukturen zur ACC (Farbduplex, *blau*)

Dem Standard in unserem Kinderkrankenhaus entsprechend wurde eine MRT-Untersuchung nach 2 Tagen durchgeführt, um die typische Panösophagitis sowie mögliche, sich darin ausbildende Fistelgänge, darzustellen. Hierbei wurde ein entzündlich-ödematös aufgetriebener Ösophagus mit gangbildenden Ulzerationen bis zur Wand der Arteria carotis communis (ACC) links gezeigt. Sonographisch konnte keine eindeutige Barriere mehr zwischen diesem Gang und der ACC dargestellt werden (Abb. 1).

Wir haben sofort eine interdisziplinäre Fallbesprechung einberufen (Kinderanästhesie, Kinderchirurgie, Kinderradiologie, Pädiatrische Intensivmedizin, Gefäßchirurgie und plastische Chirurgie). Weil bei einem akuten Anschluss der drohenden Fistel an die ACC mit einer hohen Mortalität zu rechnen war, wurde im interdisziplinären Konsens mit den Eltern die Entscheidung zu einer sofortigen operativen Exploration getroffen.

## Operation

Es erfolgte eine Querinzision zervikal links paramedian auf Höhe der vorab sonographisch markierten Ulzeration. Unter endoskopischer Kontrolle wurde der Ösophagus im Bereich des Ulkus freigelegt, die Muscularis und die Serosa waren intakt. Die anliegende ACC wurde vom Ösophagus isoliert und eine Schutzbarriere mit einem bovinen Perikard-Patch und einem Kollagenvlies eingebracht sowie endoskopisch eine transnasal positionierte Magensonde gelegt.

## Postoperativer Verlauf, Entlassung und Nachsorge

**Abb. 2:**
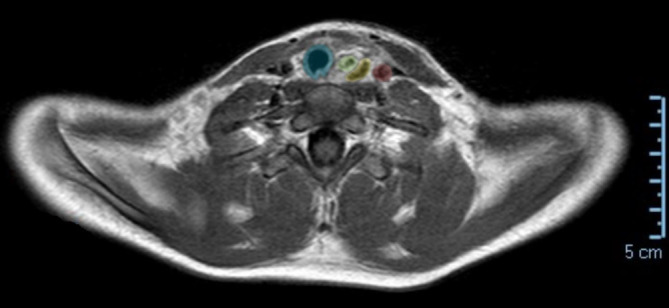
Postoperatives MRT. Trachea (*blau*), Ösophagus (*grün*) deutlich weniger wandverdickt, zwischen Ösophagus und ACC (*rot*) das Kollagenflies (*gelb*)

Die Extubation und Entfernung der Magensonde erfolgten bei klinisch und laborchemischem Normbefund bereits nach 12 h. In einem erneuten MRT am 2. postoperativen Tag war das Ödem des Ösophagus rückläufig und die operative eingebrachte Barriere zur ACC deutlich darstellbar (Abb. 2). Das Kleinkind konnte in uneingeschränktem Allgemeinzustand und nach vollständigem Kostaufbau am 3. postoperativen Tag nach Hause entlassen werden. Bei der geplanten Ösophagoskopie nach 2 Wochen waren lediglich 2 kleine narbige Areale zu sehen, welche sicher nicht obstruierend abheilen werden.

## Diskussion

In allen Industrienationen wird eine zunehmende Inzidenz schwerer Schädigungen durch die Ingestion von Knopfzellbatterien beobachtet, was sowohl durch ihre zunehmende Verbreitung als auch durch deren größere Energiekapazität erklärbar ist [4]. In der Speiseröhre löst der Stromfluss eine elektrische Hydrolyse aus, die schon nach kurzer Zeit zu schweren Kolliquationsnekrosen führt [8]. Zu den möglichen Komplikationen gehören Perforationen, tracheoösophageale Fisteln und Stimmbandparesen. Todesfälle werden v. a. durch Fisteln in Arterien berichtet [5]. Auf der ständig aktualisierten Internetseite des *National Poison Control Center* der USA werden aktuell 280 Fälle mit schwerwiegenden Komplikationen und 71 Todesfälle berichtet (Stand September 2024 [5, 6]).

Erste wichtige Sofortmaßnahme ist die sofortige Gabe von Sucralfat^1^ oder Honig^2^ [3], um die Folgen der Verätzung zu reduzieren. In verschiedenen Übersichtarbeiten [2, 3] wird diskutiert, „*bei Verletzungen nahe der Aorta*“ wiederholte MRT-Untersuchungen durchzuführen. Als Konsequenz wird das Hinzuziehen von Herz- und Thoraxchirurgie sowie eine erhöhte Bereitschaft zur einer Notfalloperation benannt. Ein klarer Rat zu einer präventiven Operation, wie in unserem Fall durchgeführt, ist in der bisherigen Literatur nicht zu finden. Weil aber ein akuter Durchbruch der gangbildenden Ulzerationen in das Gefäßsystem auch bei Ballonkatheterpositionierung im Ösophagus, transluminalen Gefäßkatheterverfahren und selbst bei sofort durchgeführter operativer Freilegung meistens tödlich verläuft [5, 7], halten wir unser Vorgehen für die einzig richtige Entscheidung in einem vergleichbaren Fall [9].

## Fazit für die Praxis

Überall, wo Kinder innerhalb der ersten 12 h nach einer Knopfzellingestion vorstellig werden, sollte unmittelbar Sucralfat oder Honig verabreicht werden. Zudem muss eine schnellstmögliche Bergung erfolgen, die aus dem oberen Ösophagus meist mithilfe eines Laryngoskops und einer geeigneten Zange gelingt. Bei sichtbaren Verätzungen des Ösophagus empfehlen wir eine MRT-Bildgebung in den Folgetagen und eine präventive operative Isolierung des entzündlichen Prozesses bei ausgeprägten Ulzera, die bedrohlich nah an Blutgefäße reichen. Zur Nachsorge sollten die Kinder daher in eine Einrichtung verlegt werden, in der die empfohlene Vorgehensweise durchgeführt werden kann. Wir empfehlen die aktualisierte Version der S2k-Leitlinie zur Fremdkörperaspiration und Ingestion bei Kindern, die zeitnah auf der AMWF-Homepage veröffentlicht werden wird.

## Footnotes

1 Sucralfat-Suspension mit 1 g/10 ml, alle 10 min 10 ml trinken bis zu 3‑mal.

2 10 ml Honig alle 10 min bis zu 6 Dosen (nicht bei Säuglingen).
